# Case Series: Genetic mimics of hypertrophic cardiomyopathy in elderly

**DOI:** 10.3389/fcvm.2025.1483390

**Published:** 2025-06-12

**Authors:** Olga S. Chumakova, Olga A. Drobyazko, Elena A. Stepanova, Alexander A. Pushkov, Kirill V. Savostyanov, Dmitry A. Zateyshchikov

**Affiliations:** ^1^ Cardiological Department, Moscow City Clinical Hospital No. 17, Moscow, Russia; ^2^ Federal Research and Clinical Center of Specialized Medical Care and Medical Technologies FMBA of Russia, Moscow, Russia; ^3^ Pathological Anatomy Department, Federal State Budgetary Educational Institution of Further Professional Education “Russian Medical Academy of Continuous Professional Education” of the Ministry of Healthcare of the Russian Federation, Moscow, Russia; ^4^ Genetic Laboratory, National Medical Research Center for Children’s Health of the Russian Federation Ministry of Health, Moscow, Russia

**Keywords:** hypertrophic cardiomyopathy, elderly, phenocopies, amyloidosis, Fabry disease, desminopathy, diagnostics, case series

## Abstract

Hypertrophic cardiomyopathy (HCM) is the most prevalent genetic cardiac disorder, primarily driven by pathogenic nucleotide variants (PNVs) in the genes that encode sarcomeric proteins. Such PNVs cause a disruption of cardiomyocytes. Notably, up to 5% of patients with an HCM phenotype may actually have other conditions that mimic HCM. These rarer, predominately hereditary syndromic diseases can be clinically suspected through specific “red flags”. However, in elderly patients, extracardiac manifestations may be subtle or misattributed to other diseases or the aging process, complicating the clinical diagnosis. In such cases, genetic testing becomes essential for achieving an accurate diagnosis and guiding specific treatment strategies. Screening younger relatives for genetic predispositions offers additional benefits in the era of emerging novel therapeutic technologies. In this study, we present comprehensive genetic and clinical characterization of three cases of HCM mimics, including amyloidosis, Fabry disease (FD), and desminopathy caused by *TTR p.V50M, GLA p.N215S,* and DES p.R355* PNVs, respectively. We also provide a brief review of the literature addressing the diagnostic challenges of associated with these rare conditions.

## 1 Introduction

HCM is the most common genetic cardiac disorder, affecting approximately 1 in 500 individuals when estimated based on overt phenotype (1). HCM is only confined to the heart, and is defined as hypertrophy of the left ventricle (LV) that cannot be attributed solely to hemodynamic loading conditions, such as systemic hypertension, aortic stenosis, or congenital heart defects (2). Classical HCM is predominantly caused by PNVs in so-called sarcomeric genes (genes encoding sarcomeric proteins). Such PNVs are identified in approximately 50% of patients (3, 4). For sarcomere-negative patients, advanced searching for missing heritability is applied (5), including testing for PNVs in the genes associated with the systemic diseases that may mimic HCM. Such mimics or phenocopies (more correctly, but rarely used, “genocopies”) comprise storage diseases (e.g., FD), infiltrative conditions (amyloidosis), mitochondrial disorders, neuromuscular diseases, and malformation syndromes (e.g., Noonan syndrome), as demonstrated by several independent studies including ours (2, 6). Although these conditions are more common in the pediatric HCM population (7), they can present later in life with subtle extracardiac manifestations. Genetic testing is a useful tool with diagnostic yield about 1.5%–5.0% for HCM mimics in adults (8–10).

Initially described in young individuals (11), HCM is now increasingly recognized in older patients, who often present with common cardiovascular risk factors and acquired conditions associated with secondary LV hypertrophy (12), complicating accurate diagnosis in this demographic. Although old age in HCM is a negative risk marker for sudden cardiac death (SCD) (13), and long-term outcomes in elderly HCM patients are comparable to those of age- and sex-matched controls (12), emerging disease-specific therapies for both sarcomeric HCM (14, 15, NCT05836259) and its phenocopies (16) - which enhance quality of life and may alter the prognosis in younger relatives – highlight the importance of accurate molecular diagnosis in old patients with LV hypertrophy.

Here, we present a case series of three 65+ HCM patients, from our prospective cohort study (17), whose diagnoses were revised following genetic testing, which included sequencing of genes associated with HCM phenotype (Supplementary Table S1). This paper describes the clinical workflows for diagnosing hereditary transthyretin amyloidosis (ATTR), FD, and desminopathy, and provides a review of the latest literature on these conditions, with a particular focus on the elderly population.

## 2 Case presentation

### 2.1 Case #1

**Figure 1:**
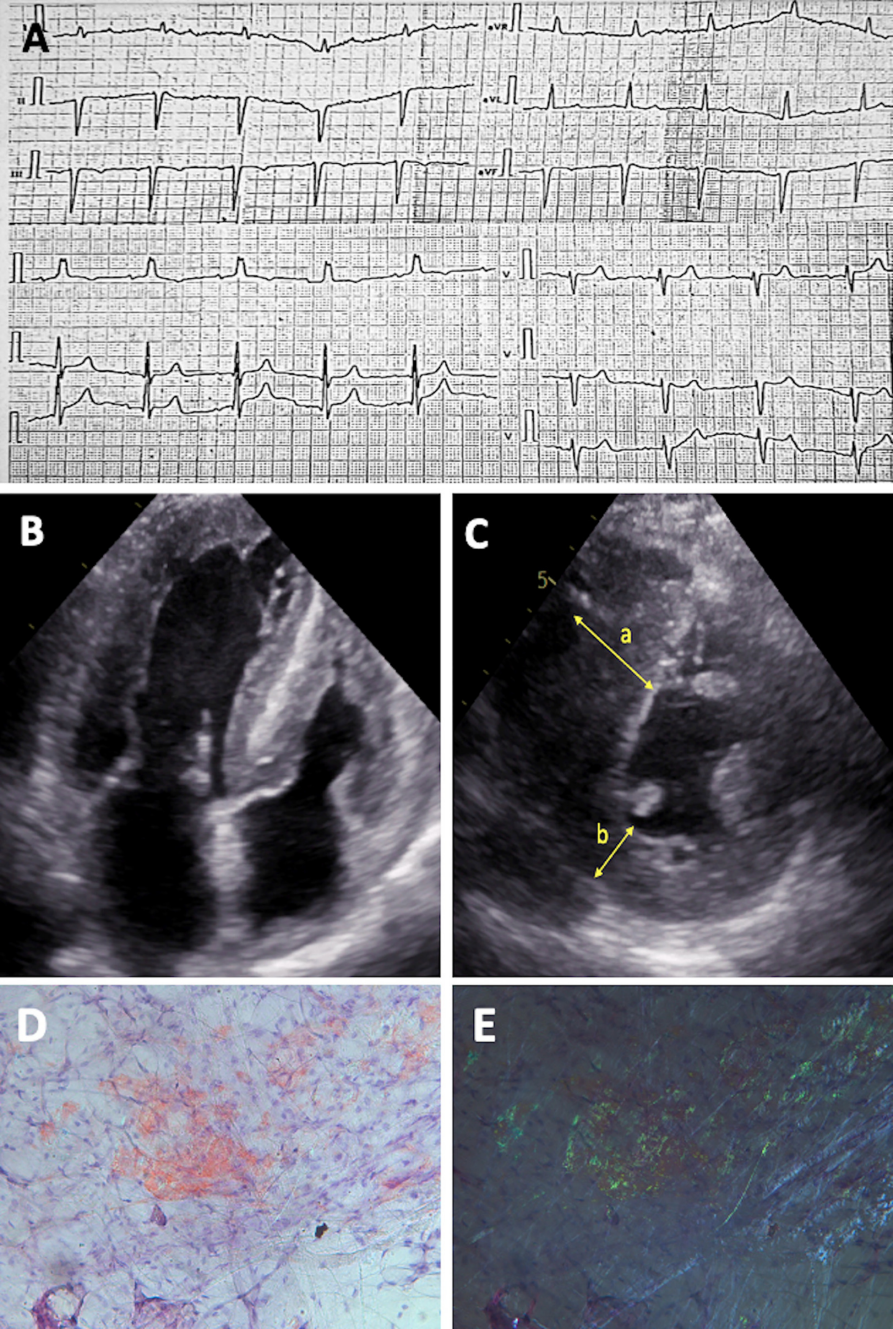
Diagnostic features of patient with hereditary ATTR amyloidosis. **(A)** ECG showing “normal” QRS voltage with left anterior hemiblock and right ventricular hypertrophy. **(B)** 2D ECHO in the apical four-chamber view and **(C)** short-axis view, revealing biventricular concentric hypertrophy with LV hypertrabeculation; measurements include interventricular septum thickness of 25 mm (a) and posterior wall thickness of 9 mm (b) **(D–E)** Histological slides of crushed subcutaneous fat tissue, stained with Congo red, at original magnification 200×. Under normal light, congophilic deposits are visible, and under polarized light, the typical birefringence of congophilic deposits is observed, graded as CR 3+ (according to B.P. Hazenberg).

A 66-year-old male patient with a history of 52 pack-years of smoking presented with a two-year history of postprandial diarrhea and weight loss. At admission, his body mass index was 22.6 kg/m^2^, and he had no chronic diseases. Initial endoscopy revealed non-specific colitis and ulcerative gastritis leading to gastroprotective therapy. Echocardiography (ECHO), prompted by electrocardiogram (ECG) changes, showed marked asymmetric LV hypertrophy, hypertrabeculation, grade 2 LV diastolic dysfunction, and right ventricular hypertrophy (Figure 1; Supplementary Video S1). Non-obstructive HCM was diagnosed incidentally since the patient had no cardiac symptoms. Holter monitoring showed no rhythm or conduction disturbances. Over the next year, the patient developed congestive heart failure and peripheral neuropathy, requiring hospitalization. Gastrointestinal signs persisted despite the therapy. Elevated serum N-terminal pro-brain natriuretic peptide (NT-proBNP) level (4,240 pg/ml) and mild renal impairment (eGFR - 76 ml/min/1.73 m^2^) were noted. Genetic testing identified a PNV in transthyretin (*TTR*) gene, NM_000371.4:*c.148G>A* (*p.V50M*), with no sarcomeric variants. Further diagnostic workup confirmed hereditary transthyretin amyloidosis (ATTR amyloidosis) through amyloid deposition in abdominal fat (Figure 1) and rectal biopsies, positive myocardial uptake on bone scintigraphy (grade 3), and the absence of monoclonal protein, which was verified by serum and urine immunofixation electrophoresis and serum free light chain assay. Familial screening was recommended (Supplementary Figure S1). Specific treatment with tafamidis was unavailable locally. Beta-blocker and diuretic therapy were initiated (Table 1). During the following year, peripheral neuropathy worsened, limiting mobility, and the patient was hospitalized for heart failure requiring parenteral diuretic therapy. Dapagliflozin and sacubitril/valsartan were initiated but discontinued soon due to hypotension.

**Table 1:** Diagnostic timeline and follow-up for elderly patients with genetic HCM mimics.

Data/signs	Case #1	Case #2	Case #3
Age diagnosis of HCM, years	66	65	18
Cardiac complaints at presentation	No	Dyspnea, angina, palpitations	Dyspnea
Cardiac signs of phenocopy beyond LV hypertrophy	“Normal” QRS voltage on ECG	Borderline LV EF	Bradycardia, Borderline LV EF
Plasma NT-proBNP, pg/ml	4,240	2,426	2,573
Extracardiac signs of phenocopy	Gastrointestinal	No	Limb weakness
Follow-up after initial diagnosis of HCM	Congestive heart failure, peripheral neuropathy	Morrow septal myectomy, pacemaker	Asymptomatic up to 59 years
Diagnostic methods used	Genetic test, extracardiac biopsy, cardiac scintigraphy, monoclonal proteins exclusion	Genetic test, enzymatic assay, electroneuromyography, ophthalmologic exam	Genetic test, creatine kinase level
Age final diagnosis, years	67	66	65
Final diagnosis	Hereditary ATTR	Fabry disease	Desminopathy
Specific treatment	Tafamidis (not available for the patient)	Agalsidase beta (was initiated)	Not exist
Common cardiac treatment with daily doses	bisoprolol 5 mg torasemide 10 mg spironolactone 50 mg	Bisoprolol 5 mg torasemide 5 mg amlodipine 5 mg Valsartan 80 mg rosuvastatin 10 mg aspirin 100 mg	Apixaban 10 mg atorvastatin 20 mg spironolactone 25 mg bisoprolol 1.25 mg
Follow-up after final diagnosis, patient's description	“I could not go out, so I moved around my apartment using a chair”	“I have lost weight and feel weak, but I can still take care of myself”	“My muscle symptoms are subtle and do not worry me”
Family genetic/clinical screening	Nephew is unaffected; Proband's offspring refused	Daughter is a carrier; granddaughter is unaffected	Family members refused

ATTR, transthyretin amyloid; ECG, electrocardiogram; EF, ejection fraction; HCM, hypertrophic cardiomyopathy; LV, left ventricular; NT-proBNP, N-terminal pro-brain natriuretic peptide.

### 2.2 Case #2

**Figure 2:**
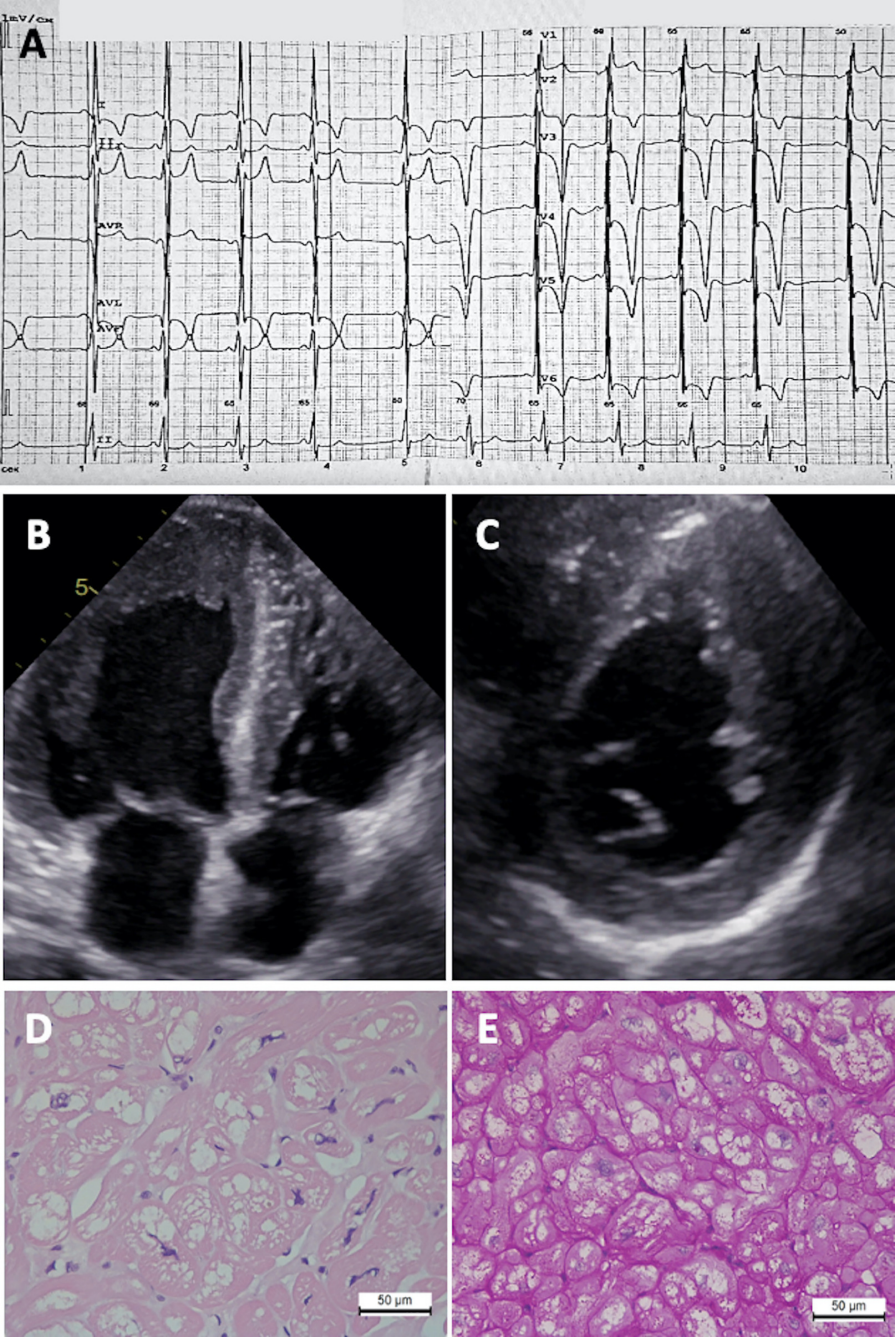
Diagnostic features of Fabry disease patient. **(A)** ECG showing LV hypertrophy, right bundle branch block, and giant T wave inversions. **(B)** 2D ECHO in apical four-chamber view and **(C)** short-axis view showing asymmetric concentric LV hypertrophy. Myocardial tissue revealing **(D)** cardiomyocyte hypertrophy and vacuolization (hematoxylin-eosin staining) and **(E)** PAS reaction.

A 66-year-old man was referred for cardiac surgery with NYHA class II dyspnea, angina, and palpitations due to severe obstructive HCM. His medical history includes moderate arterial hypertension, coronary artery disease (diagnosed at age 55), percutaneous coronary intervention for a myocardial infarction at 58, mild COVID-19 at 64, and seronegative rheumatoid arthritis. At age 65, ECG and ECHO revealed severe LV hypertrophy (Figure 2; Supplementary Video S2), and HCM with borderline normal LV ejection fraction (57%–61%) was diagnosed. Stress ECHO showed severe latent left ventricular outflow tract (LVOT) obstruction with a gradient of 107 mmHg. Holter monitoring detected no arrhythmias. Blood tests indicated elevated NT-proBNP (2,426 pg/ml) and troponin T (22.4 ng/L (≤14)) levels, along with mild renal impairment (eGFR 74 ml/min/1.73 m^2^). A Morrow septal myectomy was performed, followed by pacemaker implantation due to high-degree atrioventricular block. The patient was prescribed beta-blocker, diuretic, antihypertensive, lipid-lowering, and antiplatelet medications (Table 1). A year later, genetic testing identified a PNV in the galactosidase A (*GLA*) gene, NM_000169.3:*c.644A>G* (*p.N215S*), associated with FD, confirmed by reduced alpha-galactosidase A (α-GAL A) enzyme activity (0.66 µmol/L/h, normal > 1.89) and elevated lysosomal globotriaosylsphingosine (lyso-Gb3) level of 5.46 ng/ml (normal ≤ 2.1). No sarcomeric variants were found. Histology of myocardial tissue obtained during myectomy showed hypertrophied cardiomyocytes with extensive vacuolization (Figure 2). Electroneuromyography and ophthalmologic exams were normal. Enzyme replacement therapy (ERT) with agalsidase beta was initiated at age 67. The patient's 40-year-old daughter, a carrier of the same PNV (Supplementary Figure S1), was recommended for follow-up. After 1.5 years of ERT, the patient's condition remains stable.

### 2.3 Case #3

**Figure 3:**
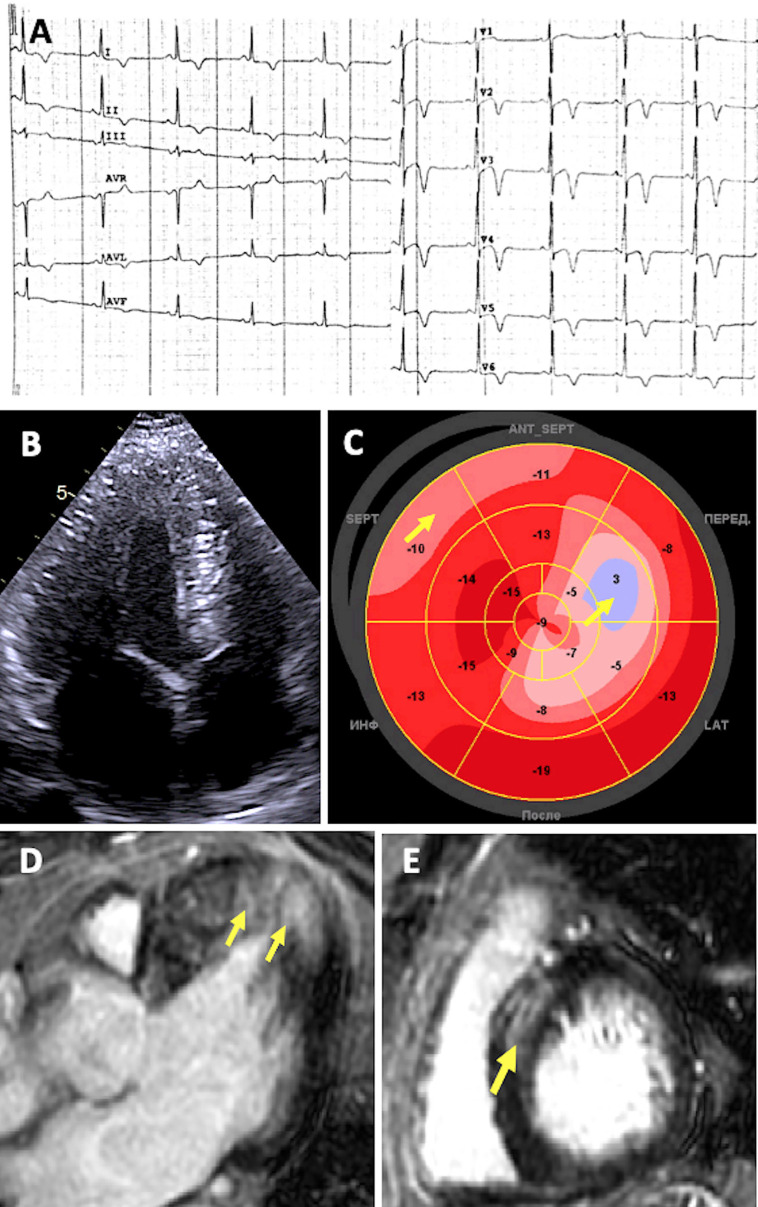
Diagnostic features of patient with desminopathy. **(A)** ECG showing sinus bradycardia and prominent repolarization abnormalities: ST segment elevation followed by deep T-wave inversions in precordial and standard leads, with a positive T wave in aVR. **(B)** 2D ECHO apical four-chamber view showing symmetric concentric LV hypertrophy. **(C)** Decreased global longitudinal strain (“bovine eye” – 11.9%) Arrows indicate more prominent localized abnormalities in systolic deformation. **(D–E)** CMR imaging showing replacement fibrosis. Arrows indicate LGE located in the same segments with decreased strain.

A 63-year-old male was presented with NYHA class II dyspnea and mild limb weakness. His medical history began at age 18, when ECG and ECHO revealed LV hypertrophy. He remained asymptomatic until age 59, when dyspnea emerged, leading to a diagnosis of non-obstructive HCM. Two years post-COVID-19, his dyspnea worsened, and limb weakness appeared. At admission, heart rate was 47 bpm; ECG was markedly changed; ECHO revealed a 23 mm LV wall thickness, grade I diastolic dysfunction, borderline LV ejection fraction of 51%, and significantly decreased LV global longitudinal strain (Figure 3, Supplementary Video S3). Cardiac magnetic resonance (CMR) imaging showed replacement fibrosis indicated by late gadolinium enhancement (LGE) (Figure 3). Holter monitoring detected frequent supraventricular (8,980) and polymorphic ventricular (560) premature beats, plus short episodes of atrial fibrillation. Biochemistry revealed elevated NT-proBNP (2,573 pg/ml), ALT (80 U/L), and troponin (74 ng/L, normal < 34.2 ng/L), with mild renal impairment (eGFR 61 ml/min/1.73 m^2^). Creatine kinase level was normal. Anticoagulant, lipid-lowering, diuretic, and beta-blocker therapy was initiated (Table 1). Family history was unremarkable (Supplementary Figure S1). Genetic testing identified a NM_001927.4:*c.1063C>T* (p.R355*) nucleotide variant in the desmin (*DES*) gene, initially classified as a variant of uncertain significance (VUS). No sarcomeric variants were found. To exclude other causes for patient's phenotype, genetic testing using a panel of over 400 genes associated with various cardiac disorders was performed, and NM_002471.4:*c.2363C>T* (*p.T788M*) VUS in the *MYH6* gene was identified. Two years later, based on novel data, the aforementioned variant in *DES* was reclassified as likely pathogenic, leading to a diagnosis of desminopathy at age 65. After one year of follow-up, the patient's cardiac and myopathy symptoms remain stable.

## 3 Discussion

### 3.1 Amyloidosis

Amyloidosis (MIM #150210) is the most common HCM phenocopy to exclude in the elderly. This condition arises from protein misfolding, leading to insoluble amyloid fibrils deposits in tissues, including the heart. Over 35 amyloidogenic protein precursors have been identified (18), with more than 95% of cardiac cases attributed to two: monoclonal immunoglobulin light chains (AL amyloidosis) and ATTR amyloidosis. The TTR, a transporter of thyroxine and retinol produced by the liver, becomes unstable due to PNVs or VUSs in the *TTR* gene or aging, thus ATTR amyloidosis can be hereditary, with autosomal dominant transmission, or wild-type (ATTRwt). Our patient (case #1) was diagnosed with hereditary ATTR amyloidosis. Estimating its prevalence is challenging due to low clinical detectability. Consequently, the prevalence of *TTR* PNVs is frequently used as a reference. The cumulative incidence of 11 *TTR* PNVs in the general population is estimated at 1 in 230, surpassing the number of diagnosed cases (19). When cardiac amyloidosis is suspected, thorough investigation of both AL and ATTR forms, following recommended guidelines, is essential (18, 20, 21).

Aside from LV hypertrophy, numerous “red flags” of cardiac and extracardiac amyloid deposition exist (20, 22), but they are often nonspecific and overlooked, delaying diagnosis by an average of 3 years (23). Our patient lacked typical ECHO features like pericardial effusion, restrictive diastole, or an apical pattern of global longitudinal strain, and had no initial heart failure symptoms or conduction abnormalities, as well as musculoskeletal or skin findings. Gastrointestinal symptoms were misattributed to alternative diagnoses, and mild renal impairment was assumed to be age-related. An ECG finding of discordantly decreased QRS voltage relative to the extent LV hypertrophy was overlooked because it did not meet the typical low-voltage criteria for amyloidosis. The later onset of peripheral sensorimotor neuropathy further delayed the correct diagnosis.

Several screening approaches exist to identify ATTR amyloidosis within the HCM population. Bone scintigraphy, with a detection rate of approximately 13%, can detect both hereditary and ATTRwt amyloidosis (24) but may yield false-negatives with in the presence of certain *TTR* mutations (25). Genetic testing for PNVs and VUSs in the *TTR* is another approach. Although ATTRwt amyloidosis is the most frequent form in individuals over 60, hereditary ATTR amyloidosis can also occur in 12%–15% of cases (23, 26).

Over 140 *TTR* variants have been identified (source: amyloidosismutations.com, accessed on 10AUG2024). The *c.148G>A:p.V50M* (formerly *p.V30M*), identified in our patient, is the first described (27) and most prevalent worldwide. Most carriers are clustered in endemic regions of Portugal and Sweden, where it typically manifests as a peripheral neurological phenotype (9). Unlike variants such as *p.V142I*, *p.T80A*, or *p.I88l*, the *p.V50M* is not associated with a predominant cardiac phenotype (9). Cardiac involvement in *p.V50M*-associated amyloidosis is observed in 20% of patients under 50 years, 35% over 50 (9), and 23% over 70 years (26). The clinical heterogeneity, common in Mendelian diseases like ATTR amyloidosis, is increasingly attributed to non-coding genetic variants affecting gene expression (28).

In our 65-year-old patient from a non-endemic region, ATTR amyloidosis was identified through genetic testing due to an asymptomatic HCM phenotype, though gastrointestinal symptoms were initially misdiagnosed. Gastrointestinal manifestations are particularly common in hereditary ATTR amyloidosis, especially with *p.V50M* variant (29). Our case underscores the diagnostic challenges of this rare condition, which often requires a multi-specialist approach. Specific therapies of ATTR amyloidosis are rapidly developed. Tafamidis, the first ATTR-stabilizer, is now approved for clinical use. New therapeutic classes are in late-stage clinical trials, including silencers, antibodies and gene therapy (30). Based on prognostic staging (9) and lack of specific treatment, the patient's life expectancy is less than three years.

### 3.2 Fabry disease

FD (MIM #301500) is an X-linked lysosomal storage disorder caused by PNVs in the *GLA* gene, leading to reduced α-GAL A enzyme activity. This deficiency results in the accumulation of glycosphingolipids, primarily globotriaosylceramide (Gb3) and its deacylated form, lyso-Gb3, inside lysosomes, causing multiple organ dysfunction (31). In LV hypertrophy/HCM cohorts, 0.4%–1.0% of cases are actually FD (17, 32–34). FD diagnosed at age 65+ is rare (<8% of cases) (35), which reduces practitioner alertness and complicates early detection and specific treatment, such as ERT. In unselected UK Biobank population, the prevalence of later onset Fabry-causing *GLA* variants is 1 in 5,732, higher than the reported prevalence of FD (36), indicating the disease is underdiagnosed.

FD includes a classical form in pediatric and adolescent males and females with neurological pain, angiokeratoma, gastroenterological, and ophthalmological symptoms (37). An atypical late-onset form, usually diagnosed in males aged 30–70, often presents as LV hypertrophy that can mimic HCM (21, 38, 39). This form is linked to residual α-GAL A enzyme activity, which protects endothelial and smooth muscle cells, but not cardiomyocytes, from glycosphingolipid accumulation (40, 41). This selective deposition in cardiomyocytes triggers chronic inflammation, a key pathogenic factor in FD-related cardiomyopathy (42).

The patient in case #2 had a 34% residual α-GAL A enzyme activity and exhibited late-onset FD. He presented with symptomatic HCM related to latent LVOT obstruction, seen in approximately 40% of FD patients with an HCM phenotype along with exertional cardiac symptoms (43). FD should be considered in the differential diagnosis through cardiac manifestations beyond LV hypertrophy, such as conduction disturbances, arrhythmias, chest pain secondary to microvascular ischemia, and valvular dysfunction (35, 44, 45). FD patients have a 25-fold increased incidence of anti-bradycardia pacemaker implantation (46) compared to the general population. However, our patient lacked these features and received a pacemaker due to complications following surgery. In the presence of coronary artery disease, angina could not be attributed to microvascular ischemia without further examination. FD is associated with some ECG changes, including right bundle branch block (RBBB), short PQ interval, prolonged QRS interval, R wave in lead aVL ≥ 11 mm, and inferior ST depression. RBBB, presented in our patient, is observed in approximately one-third of FD patients, compared to only 6% of those with HCM, making it a particularly distinguishing ECG feature of FD (47, 48).

CMR imaging is the optimal non-invasive modality for diagnosing FD, particularly in the presence of LV hypertrophy or early cardiac involvement. FD patients typically demonstrate LGE in the basal inferolateral wall (49), a strong predictor of FD in HCM cases, especially when combined with a bifascicular block on ECG (39). Low native T1 mapping is another distinctive CMR marker that helps distinguish FD from LV hypertrophy of other origins (50). In FD, LGE is often accompanied by chronic T2 mapping elevation, indicating localized edema, and is associated with persistent troponin elevation, observed in 38% of FD patients (51). Although CMR was not performed due to the pacemaker, our patient had elevated plasma troponin level, suggesting underlying myocardial fibrosis and edema.

Contrary to females, in males FD can be distinguished from HCM using enzymatic assays or genetic testing as a first-line diagnostic tool (39). Our case illustrates that even with a known PNV in *GLA*, FD signs can be subtle. Therefore, using genetic panels that include the *GLA* gene, followed by enzymatic testing, is optimal for diagnosing late-onset FD. Identifying disease in relatives is crucial, given the advantages of early administration of approved therapies like ERT or chaperones, as well as the potential for emerging therapies, including plant-derived ERTs, substrate reduction, mRNA therapies, and gene editing (52). We identified one asymptomatic *p.N215S* variant carrier (the patient's daughter), who will be monitored, while the patient's son was not genetically tested due to the X-linked mode of FD transmission. Our patient began ERT at age 66. Although the effectiveness of ERT in elderly patients remains uncertain, it may prevent LV hypertrophy progression and associated complications (53–55).

As with other inherited cardiomyopathies, FD phenotype cannot be predicted by genotype alone, but specific *GLA* variants, like *p.N215S*, are associated with late-onset cardiac FD (38, 39, 56, 57). Carriers of the *p.N215S* typically exhibit later onset of symptoms (57 vs. 9 years), LV hypertrophy (64 vs. 41 years), and proteinuria (71 vs. 43 years), with lower lyso-Gb3 levels (6.7 vs. 74.3 nmol/L) and greater survival rates (81 vs. 66 years) (38, 58). Despite the fact that our patient fits the profile of a typical *p.N215S*-associated FD case, this condition is an orphan disease, making timely diagnosis challenging, particularly in elderly patients where subtle extracardiac features are often attributed to age-related processes (56).

### 3.3 Desminopathy

PNVs in the *DES* gene cause various skeletal and cardiac muscle disorders known as desminopathy (MIM #125660). The desmin protein belongs to the muscle-specific type III intermediate filament (IF) family, which connects different cell organelles and multi-protein complexes, providing structural integrity and flexibility of myocytes (59). Affected IF networks result in insoluble granulo-filamentous material accumulation in muscle cells.

A meta-analysis of 159 *DES* mutation carriers with 40 different mutations showed that 22% had isolated cardiac signs, 22% had isolated neurological signs, and 49% had both; over 70% experienced skeletal myopathy or muscular weakness, with normal creatine kinase levels in one third of them; 60% had cardiac conduction issues or arrhythmias; and up to 50% had cardiomyopathy (60). *DES* variants are associated with dilated, arrhythmogenic (61, 62), and restrictive cardiomyopathies (63). HCM - the rarest manifestation of DES myopathy – was also observed (21). Proteomic studies on myocardial samples from both HCM mice and patients have shown increased levels of desmin (64–67), suggesting that desmin may play a role in impairing the ubiquitin-proteasome system in HCM (67). Recently, the ClinGen Hereditary Cardiovascular Disorders Gene Curation Expert Panel (HCVD-GCEP) confirmed *DES* as definitively associated with the HCM phenotype (68).

In our patient (case #3), HCM first appeared in his 20s without the typical signs of desminopathy, such as atrioventricular block and skeletal myopathy, which only became evident in his 60s when muscle symptoms emerged. He had elevated troponin levels but normal creatine kinase levels in his blood – a pattern also noted in another DES-HCM case (69).

To date, over 160 rare variants in *DES* are considered pathogenic (accessed on 14AUG2024 from https://franklin.genoox.com/clinical-db/gene/hg19/DES), and only a few cases of HCM associated with *DES* variants have been reported (Supplementary Table S2). These cases were confirmed by cytoplasmic desmin-positive immunoreactivity, a hallmark of desminopathy. The DES p.R355* variant identified in our patient has been entered in ClinVar four times in association with “dilated cardiomyopathy”, “cardiac manifestations”, and “desmin-related myofibrillar myopathy”. It was also identified as a secondary actionable finding in a whole exome sequencing cohort (70). However, no clinical data were provided. Located in exon 6, this C to T substitution at nucleotide position 1,063 Argining to stop codon, and results in the synthesis of a truncated protein. Truncating variants in *DES* have been validated as definitive causes of HCM by ClinGen. Consequently, despite the absence of a biopsy, we classified the p.R355* variant as likely pathogenic in case #3, characterized by early-onset LV hypertrophy, late heart failure progression, and mild myopathy.

As shown in case #3, genetic cardiomyopathies can have an asymptomatic course lasting decades, beginning at a young age. Earlier recognition of such cardiomyopathies is crucial, as not all patients survive to an advanced age. Participation in sports, which is common amongst the young, further increases the risk of SCD in these individuals. ECG appears to be the most valuable tool for early detection, often revealing abnormalities such as repolarization or depolarization disturbances well before structural changes in the heart occur. Another important ECG finding is the presence of frequent premature ventricular complexes (PVCs) in young adults, in whom coronary artery disease is not usually anticipated. While PVCs are generally considered benign in asymptomatic young individuals, they may indicate an underlying rare cardiomyopathy that poses a high risk of SCD (71). In adult patients, the burden of PVCs may also provide alerts for disease progression at the molecular level (72).

## 4 Conclusion

The rarity of Mendelian disorders and the presence of age-related conditions that can mimic the clinical presentation of the disease make it challenging to diagnose HCM phenocopies in elderly. At the same time, specific treatments for some HCM phenocopies are currently available and can significantly improve the quality of life for elderly patients. Routine genetic testing in this demographic is crucial for establishing an accurate diagnosis, which is even more important for their younger relatives who may benefit from emerging therapies, such as gene therapies.

## Data Availability

The datasets presented in this study can be found in online repositories. The names of the repository/repositories and accession number(s) can be found in the article/Supplementary Material.

## Supplementary material

Supplementary Video S1 Transthoracic echocardiography of patient with hereditary ATTR amyloidosis, apical four chamber view.

Supplementary Video S2 Transthoracic echocardiography of patient with Fabry disease, apical four chamber view.

Supplementary Video S3 Transthoracic echocardiography of patient with desminopathy, apical four chamber view.
