# Supplementary material for: Case Series: Genetic mimics of hypertrophic cardiomyopathy in elderly
Source: Front Cardiovasc Med. 2025 Jun 12;12:1483390. doi: 10.3389/fcvm.2025.1483390 (PMC12198175; doi:10.3389/fcvm.2025.1483390)
Supplement: Supplementary file 4 [file Datasheet1.docx]

Supplementary Material

# Supplementary Table S1. Genes sequenced and analyzed in all presented patients

| **Gene name** | **Coding protein name** |
| --- | --- |
| *ACTC1* | Alpha-actin |
| *DES* | Desmin |
| *FLNC* | Filamin C |
| *GLA* | Alpha-galactosidase A |
| *LAMP2* | Lysosome-associated membrane protein 2 |
| *MYBPC3* | Myosin binding protein C |
| *MYH7* | Myosin heavy chain 7 |
| *MYL2* | Regulatory light chain |
| *MYL3* | Essential light chain |
| *PLN* | Phospholamban |
| *PRKAG2* | 5’-AMP-activated protein kinase subunit gamma-2 |
| *PTPN11* | Tyrosine protein phosphatase non-receptor type 11 |
| *TNNC1* | Troponin C |
| *TNNI3* | Troponin I |
| *TNNT2* | Troponin T |
| *TPM1* | Alpha-tropomyosin |
| *TTR* | Transthyretin |

**Supplementary Table S2. Clinical and genetic data on previously reported HCM cases associated with variants in *DES* gene**

|  | **Sex / Origin** | **Age of HCM onset** | **Age at final diagnosis** | **Genetic variant** | **HCM characteristics** | **Other cardiac signs** | **Skeletal muscle signs** | **Outcome** | **Ref** |
| --- | --- | --- | --- | --- | --- | --- | --- | --- | --- |
| 1 | Male / Russian | 15y | 27y | *c.735+1G>A* | Enlarged LA, LV WT transferred from 17 to 13mm, moderate PE, LGE | Frequent VPB, NSVT, 2nd AVB, elevated Tn level | No | Pacemaker, ICD | (69) |
| 2 | Male / Chinese | 9y | 15y | *c.1216C > T, p.R406W* | Enlarged both atria, thickened IVS | AF, ABBB | Mild | Not provided | (73) |
| 3 | Male / Japanese | 9y | 28y | *c.1216C > T*, *p.R406W* | HCM progressed to the end stage with LV EF 25% | AVB | Mild | VAD | (74) |
| 4 | Female / Japanese | 7y | 8y | *c.1216C > T*, *p.R406W* | IVS 15.5mm | RBBB, complete AVB | No | ICD | (75) |
| 5 | Male / Japanese | 13y | 18y | *c.655A>C, p.T219P*  (Homozygote) | Bi-ventricular hypertrophy, RA hypertrophy. Progressed to enlarged LV and thin WT | AF, CHF, RBBB, wide QRS tachycardia | Progressive | Sudden death at 20y | (76) |
| 6 | Male / Russian | 18y | 65y | *c.1063C>T,*  *p.R355** | Severe concentric LVH (23mm); LV EF 51% LGE | Bradycardia, frequent VPB, AF, elevated troponin level | Mild | HF | Present case |

ABBB – altering bundle branch block; AF – atrial fibrillation; AVB – atrioventricular block; CHF – congestive heart failure; EF – ejection fraction; HCM – hypertrophic cardiomyopathy; HF – heart failure; ICD – implanted cardioverter-defibrillator; IVS – interventricular septum; LA – left atrium; LGE – late gadolinium enhancement; LV – left ventricular; LVH – left ventricular hypertrophy; NSVT – non-sustained ventricular tachycardia; PE – pericardial effusion; RBBB – right bundle branch block; Tn – troponin; VAD – ventricular-assisted device; VPB – ventricular premature beats; WT – wall thickness

**Supplementary Figure 1 .** Pedigrees for patients diagnosed with HCM phenocopies in old age. (A) Hereditary ATTR amyloidosis case (*TTR* *c.148G>A:p.V50M*); (B) Fabry disease case (*GLA* *c.644A>G:p.N215S*); (C) Desminopathy case (*DES* *c.1063C>T:p.R355**). Arrows indicate probands; solid symbols: affected; N: not affected; vertical bar inside symbol: asymptomatic carrier; squares: males; circles: females; rhombuses: both

**References for Table S2:**

69. Gudkova A, Kostareva A, Sjoberg G, Smolina N, Turalchuk M, Kuznetsova I, et al. Diagnostic challenge in desmin cardiomyopathy with transformation of clinical phenotypes. Pediatr Cardiol. (2013) 34(2):467–70. doi: 10.1007/s00246-012-0312-x

73. Xiao H, Song L, Tao L. A case report of adolescent myofibrillar myopathy due to a *de novo* R406W pathogenic variant in desmin with symptoms of "hypertrophic cardiomyopathy". *Heliyon*. (2024) 10(3):e25009. doi: 10.1016/j.heliyon.2024.e25009

74. Takegami N, Mitsutake A, Mano T, Shintani-Domoto Y, Unuma A, Yamaguchi-Takegami N, et al. The Myocardial Accumulation of Aggregated Desmin Protein in a Case of Desminopathy with a de novo DES p.R406W Mutation. *Intern Med*. (2023) 62(19):2883-2887. doi: 10.2169/internalmedicine.0992-22

75. Oka H, Nakau K, Imanishi R, Furukawa T, Tanabe Y, Hirono K, et al. A Case Report of a Rare Heterozygous Variant in the *Desmin* Gene Associated With Hypertrophic Cardiomyopathy and Complete Atrioventricular Block. *CJC Open*. (2021) 3(9):1195-1198. doi: 10.1016/j.cjco.2021.05.003

76. Harada H, Hayashi T, Nishi H, Kusaba K, Koga Y, Koga Y, et al. Phenotypic expression of a novel desmin gene mutation: hypertrophic cardiomyopathy followed by systemic myopathy. *J Hum Genet.* (2018) 63:249–254. <https://doi.org/10.1038/s10038-017-0383-x>

# Data Availability Statement

The DNA-seq datasets for this study can be found in the Sequence Read Archive (SRA) under the accession number PRJNA1149685 (<https://www.ncbi.nlm.nih.gov/bioproject/PRJNA1149685>).
