# Supplementary figures and images for: Case Series: Genetic mimics of hypertrophic cardiomyopathy in elderly
Source: Front Cardiovasc Med. 2025 Jun 12;12:1483390. doi: 10.3389/fcvm.2025.1483390 (PMC12198175; doi:10.3389/fcvm.2025.1483390)

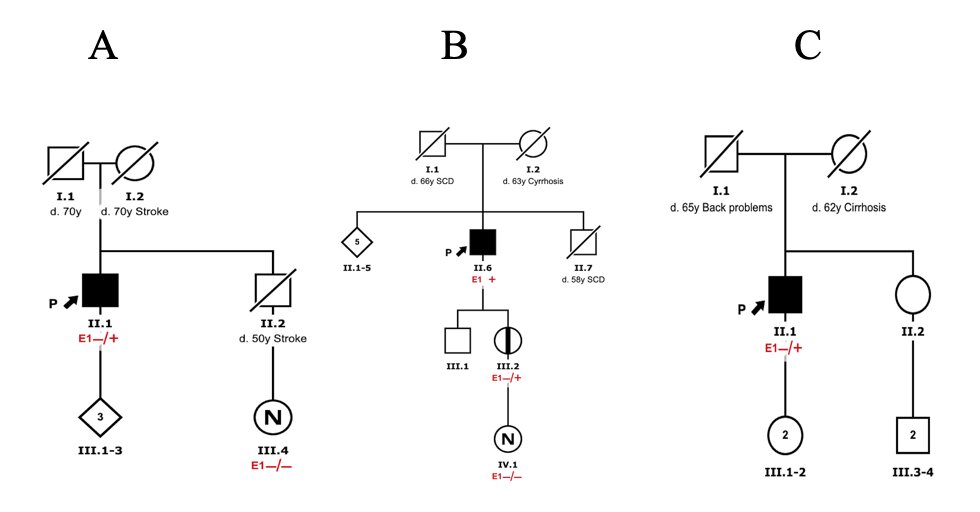

Supplement: Supplementary file 5 [file Image1.jpeg]
